# Activity-Dependent Callosal Axon Projections in Neonatal Mouse Cerebral Cortex

**DOI:** 10.1155/2012/797295

**Published:** 2012-11-19

**Authors:** Yoshiaki Tagawa, Tomoo Hirano

**Affiliations:** ^1^Department of Biophysics, Kyoto University Graduate School of Science, Kitashirakawa-Oiwake-cho, Sakyo-ku, Kyoto 606-8502, Japan; ^2^CREST, Japan Science and Technology Agency, Kawaguchi, Saitama 332-0012, Japan

## Abstract

Callosal axon projections are among the major long-range axonal projections in the mammalian brain. They are formed during the prenatal and early postnatal periods in the mouse, and their development relies on both activity-independent and -dependent mechanisms. In this paper, we review recent findings about the roles of neuronal activity in callosal axon projections. In addition to the well-documented role of sensory-driven neuronal activity, recent studies using in utero electroporation demonstrated an essential role of spontaneous neuronal activity generated in neonatal cortical circuits. Both presynaptic and postsynaptic neuronal activities are critically involved in the axon development. Studies have begun to reveal intracellular signaling pathway which works downstream of neuronal activity. We also review several distinct patterns of neuronal activity observed in the developing cerebral cortex, which might play roles in activity-dependent circuit construction. Such neuronal activity during the neonatal period can be disrupted by genetic factors, such as mutations in ion channels. It has been speculated that abnormal activity caused by such factors may affect activity-dependent circuit construction, leading to some developmental disorders. We discuss a possibility that genetic mutation in ion channels may impair callosal axon projections through an activity-dependent mechanism.

## 1. Introduction

For developmental neuroscientists, interhemispheric axons (callosal axons) have been an excellent model to study how long-range axonal projections develop in the brain. Callosal axons form one of the major axonal tracts in the mammalian brain, the corpus callosum, which visibly connects the two cerebral hemispheres. In the past decades, detailed anatomical and physiological studies in animal models have improved our understanding of the organization and development of callosal connections [1–8]. Recent genetic studies have revealed molecular signals critical for the identity specification of callosal projection neurons [9–12] and axon guidance during the midline crossing [13–27]. These findings have been relevant for not only basic neuroscientists but also clinical neuroscientists, because malformations such as partial or complete agenesis of the corpus callosum are associated with many human congenital disorders [18, 22].

In general, during formation of connections in the developing brain, there is an early phase relying on activity independent mechanisms (such as those involving axon guidance molecules) and a later phase requiring neuronal activity [28–33]. As for callosal connections, an important role of neuronal activity in their formation is well established. For example, in the visual cortex, sensory-driven neuronal activity is crucial for the formation of callosal connections [7, 34–37]. In addition, recent studies using mice as a model have begun to reveal critical roles of neuronal activity spontaneously generated in neonatal cortical circuits [38–41]. Sophisticated in vivo Ca^2+^ imaging and multiunit recordings have uncovered several distinct patterns of neuronal activity occurring in neonatal mouse cortex during the periods when callosal axon projections develop [42–50]. Interestingly, some of the activities occurring in both hemispheres are synchronized [47]. These new findings suggest that callosal axon projections and connection formation are shaped not only by sensory-driven neuronal activity but also by spontaneous neuronal activity generated in the developing cerebral cortex.

In this paper, we focus on activity-dependent mechanism of callosal projection formation. First, we review recent findings about the role of spontaneous neuronal activity in callosal axon projections. We then summarize the patterns of neuronal activity observed in the developing cerebral cortex, which might play a role in activity-dependent callosal axon projections and cortical circuit formation in general. Finally, we discuss a possibility that abnormal neuronal activity caused by genetic mutations in ion channels might influence activity-dependent phases of callosal axon projections, leading to some deficit in the structure/function of the corpus callosum.

## 2. Activity-Dependent Development of Callosal Axon Projections in the Mouse

**Figure 1:**
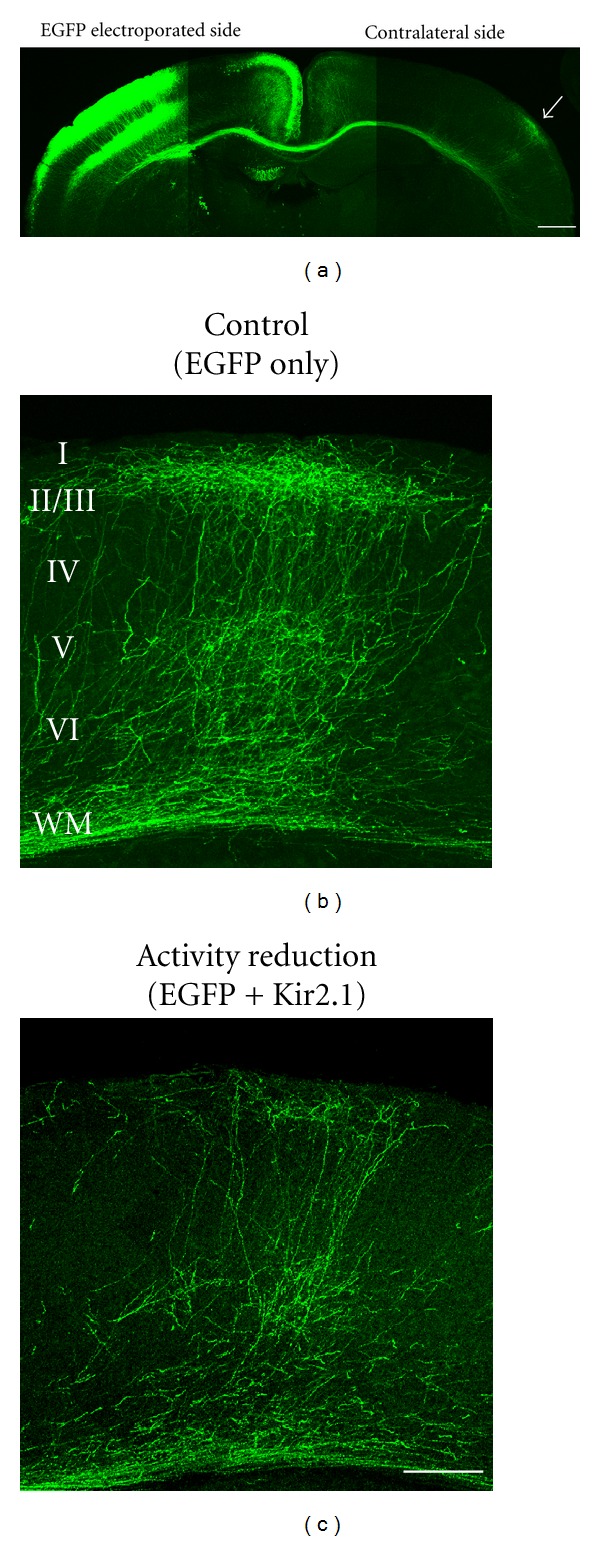
Visualization of callosal axon projections and an effect of activity reduction. (a) An in utero electroporation-mediated gene transfer method [38] was used to unilaterally express EGFP in layer 2/3 cortical neurons (electroporated side). EGFP-labeled callosal axons extend through the corpus callosum, and project densely to a narrowly restricted region in the contralateral cortex (arrow). Scale bar, 500 *μ*m. (b) EGFP-labeled control callosal axons show lamina specific projection pattern. (c) Reduction of neuronal activity in callosal projection neurons disturbs their axonal projections. Adapted from [38]. Scale bar, 200 *μ*m.

Callosal axons are derived from cortical layer 2/3 and 5 neurons [51]. During development, they extend down towards the white matter, turn medially towards the midline, run in the white matter, cross the midline, extend through the white matter of the contralateral cortex, locate their target neocortical area for innervation, turn and make terminal arbors and synapses in the correct cortical layers (Figure 1). The formation of such long-range axonal projections could rely on activity independent and activity-dependent mechanisms. To test if neuronal activity is required for callosal axon projections, and, if it is, to determine which stage(s) of axonal development is activity-dependent, we examined the development of callosal axons in mouse visual cortex under the condition where the activity of callosal projection neurons was reduced [38]. We used a genetic technique of in utero electroporation for labeling callosal axons with EGFP while reducing the activity of callosal projection neurons with a potassium channel Kir2.1, a genetic tool to reduce neuronal activity [52, 53]. In control animals in which only EGFP was electroporated unilaterally at embryonic day 15 (E15), EGFP was expressed in layer 2/3 cortical excitatory neurons, and EGFP-labeled callosal axons extending from the electroporated hemisphere to the other were observed (Figure 1). In the visual cortex contralateral to the electroporated side, EGFP-labeled callosal axons projected densely to a narrowly restricted region at the border between the primary and secondary visual cortex, in which they terminated mostly in layers 1–3 and 5, and less in layers 4 and 6 (Figure 1(b)). This region-and layer-specific pattern of projection is consistent with the pattern observed in earlier studies using dye tracers [5, 54]. When Kir2.1 was electroporated with EGFP in layer 2/3 cortical excitatory neurons, their firing rate was reduced [38] as expected from earlier studies [52, 53, 55]. The effect of Kir2.1 expression on callosal axon projections was robust: terminal arborization of EGFP-labeled callosal axons especially in layers 1–3 was dramatically reduced (Figure 1(c)). In contrast, their midline crossing and extension to the target innervation area appeared unaffected. These results suggest that early phases of callosal axon development do not require neuronal activity, but that late phases (e.g., growth of axons and their arbors within the innervation area) are activity-dependent [38, 56]. 

Wang and colleagues took a similar approach to examine the role of neuronal activity in callosal axon projections in the somatosensory cortex [40]. Suppressing excitability of callosal projection neurons by Kir2.1 expression reduces arborization of callosal axons at the border region between the primary and secondary somatosensory cortex, with some aberrant projections radially and tangentially. In addition, they used tetanus toxin light chain (TeNT-LC), a genetic tool to block transmitter release from axon terminals, to show that blocking synaptic transmission also affects callosal axon projections. Interestingly, TeNT-LC expression causes more severe effects on callosal axon projections than Kir2.1 expression: blocking synaptic transmission via TeNT-LC expression produces a more pronounced reduction in the projections to the target cortical layers, and the eventual loss of callosal projections. These results suggest that neuronal and synaptic activities are critically involved in callosal axon projections in the somatosensory cortex.

**Figure 2:**
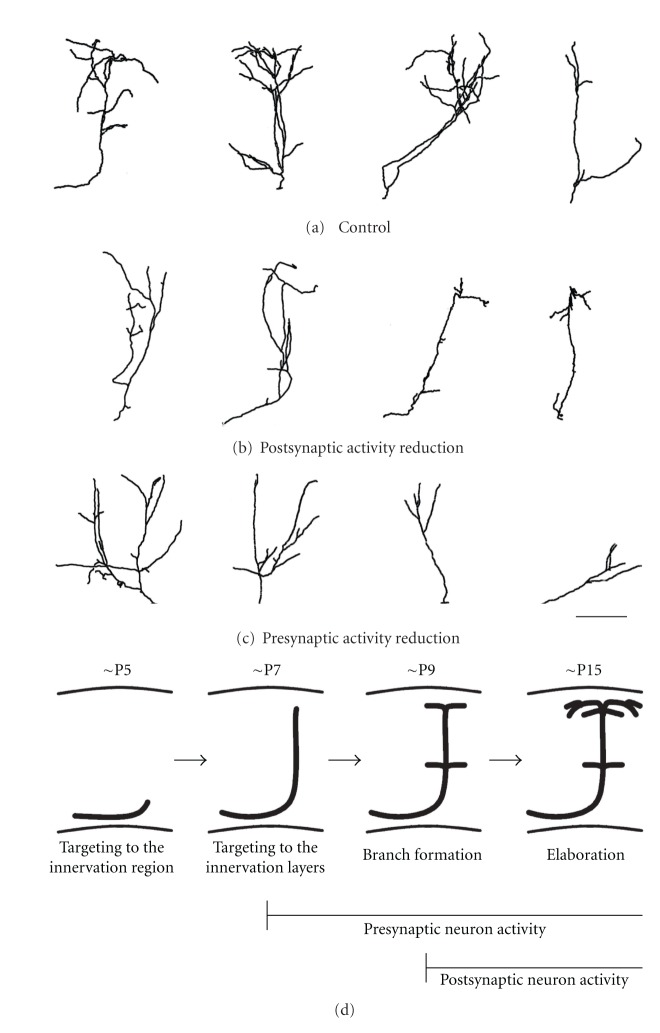
Effects of postsynaptic and presynaptic activity reduction on callosal axon projections. (a) The morphology of single callosal axons in the normal condition at P15. ((b) and (c)) postsynaptic (b) and presynaptic (c) activity reduction impede growth and branching of callosal axons. Scale bar, 200 *μ*m. (d) An illustration showing the development of callosal axons in the mouse. Callosal axons reach the target innervation area around P5, arrive in the target cortical layers at P7, start to branch at P9, and elaborate their arbors afterwards. The effect of presynaptic activity reduction is apparent before axons reach the target cortical layers, but that of postsynaptic activity reduction is observed after their arrival in the target layers. Adapted from [41].

Many studies have suggested that the formation of connections requires both presynaptic and postsynaptic neuronal activities [28, 29]. To test if postsynaptic neuronal activity is required for callosal axon projections, we performed more intricate electroporation experiments in which one side of the cortex was electroporated with EGFP for labeling single callosal axons while the other side was electroporated with Kir2.1 for postsynaptic neuron silencing [41]. We found that callosal axons under postsynaptic activity reduction appeared normal until they contacted the putative postsynaptic neurons. However, callosal axons under postsynaptic activity reduction remained less branched after they reached the target cortical layers (Figure 2(b)). This was in contrast with control callosal axons that showed extensive branching (Figure 2(a)). These results suggest that postsynaptic neuronal activity is required for arborization of presynaptic axons after these axons contact the postsynaptic neurons (Figure 2(d)). Axon arbor growth may be enhanced by synaptogenesis [57, 58]; an axon arbor making stable synapses may grow further, while that making less stable synapses may not be able to grow and eventually retract. Formation and maturation of synapses require coordinated presynaptic and postsynaptic activity [28, 29]. If either (or both) were reduced, synapse formation would be suppressed, which may lead to poor arborization of presynaptic axons. This transsynaptic effect may be mediated by some retrograde signal(s) from postsynaptic neurons to presynaptic axons. Possible candidates would be neurotrophins, which are shown to act as retrograde messengers in mediating activity-dependent strengthening of synaptic connections [59–63].

We also found that the effect of presynaptic neuronal activity reduction was apparent before axons reached the target cortical layers (Figures 2(c) and 2(d)) [41]. This result suggests that the activity of projection neurons themselves plays a role in axonal development before synapse formation. How does activity of callosal projection neurons regulate their own axonal development? Neuronal activity may modulate cytoskeleton rearrangement in the growing axons. Ohnami and colleagues have found that RhoA, a member of Rho family small GTPases, acts as a positive regulator for activity-dependent axon branching in cortical neurons [64]. It is also shown that neuronal activity can modulate the expression or function of some guidance molecules expressed on growing axons, thereby regulating axonal growth, pathfinding, fasciculation, and branching [65–68].

What intracellular signaling mediates activity-dependent axonal development? Kir2.1 overexpression in cortical neurons hyperpolarizes their membrane potential and increases the threshold for evoking action potentials, thereby inhibiting the firing activity [38, 40, 52]. This reduction in firing could attenuate intracellular Ca^2+^ signaling. It is known that Ca^2+^ plays a critical role in the regulation of neuronal morphogenesis including dendrite and axon development [69–73]. There are many protein kinases and phosphatases whose activities are regulated by Ca^2+^. Ageta-Ishihara and colleagues showed that a member of the Ca^2+^-dependent kinase family, Ca^2+^/calmodulin-dependent protein kinase I*α*  (CaMKI*α*), plays a critical role in callosal axon projections [74]. Using in vitro dissociated culture system, they found that blocking expression or function of CaMKI*α* specifically impaired axonal, but not dendritic, growth of cortical neurons. They also found that activation of GABA_A_ receptors promoted axonal growth in a CaMKI*α*-dependent manner. They further showed that in vivo RNAi knockdown of CaMKI*α* in callosal projection neurons by in utero electroporation disturbed their axonal projections. It is known that the action of GABA is excitatory in the neonatal period (until two weeks of age) [75]. Although it was not shown whether GABA exerted its action on the cell body and dendrites, or directly on the growing axons, their results suggest that CaMKI*α* is critically involved in activity-dependent callosal axon projections and that this activity is at least in part mediated by excitatory action of GABA. 

Many issues remain to be addressed. For example, the work of Ageta-Ishihara and colleagues suggests that CaMKI*α* is an important player which may work downstream of neuronal activity, but other possible candidates (Ca^2+^-dependent and independent intracellular signaling molecules) remain unexplored. In addition, these intracellular signaling molecules would influence the regulation of cytoskeletal proteins, thereby regulating growth and branching of axons, but the precise molecular mechanism is unknown. It is important to note that some of intracellular signaling molecules might work both activity dependently and independently: for example, they may be involved in midline crossing of callosal axons under the control of some guidance molecules and subsequently play a role in axon arbor growth and branching under the control of neuronal activity. If this is the case, intricate experiments such as those using temporally controlled RNAi knockdown of target molecule would be necessary.

Another important issue to be addressed would be the possible relationship between the process of thalamocortical projections and that of the formation of callosal connections. In the visual cortex during the neonatal period, the activity of cortical neurons is modulated by thalamocortical inputs, which transmit activity from the periphery (spontaneously generated massive neuronal activity in the retina, called retinal waves) [50, 76]. Thalamocortical connectivity develops until P8 in the mouse [77], several days before callosal connectivity forms [38]. Is thalamocortical innervation a prerequisite for callosal connections to establish? Does the activity supplied by thalamocortical inputs play a role in callosal connection formation? These are important not only from a developmental point of view but also from a functional view. Thalamocortical projections are arranged in the cortex in a retinotopic manner, and each visual callosal axon projects to a retinotopically matched region within the visual cortex [78–81]. Olavarria et al. have shown that eye removal during the neonatal period alters retinotopically matched projection pattern of callosal axons [54, 82], suggesting a possibility that retinotopic information conveyed through the retino-thalamo-cortical pathway influences callosal connection formation. It would be interesting to examine whether eliminating thalamocortical projections (or suppressing activity of thalamocortical axons) also affects retinotopically organized callosal projection pattern. In addition, whether callosal axons under activity reduction (such as those shown in Figures 2(b) and 2(c)) show retinotopically correct projection pattern or not would be an important issue to be addressed in future.

## 3. Patterns of Neuronal Activity Observed ****in Rat/Mouse Cerebral Cortex during ****the Neonatal Period

Recent studies have revealed that several distinct patterns of neuronal activity take place in the rat/mouse cortex during the periods when callosal axon projections develop [42–50, 83–85]. Some of them are asynchronous (i.e., neurons fire action potentials individually), and others are network events in which activities of many neurons are synchronized locally or globally (called “cortical waves”). It is important to note that neurons in the sensory cortex can fire action potentials without sensory inputs. In development, cortical neurons differentiate to express a combination of ion channels, by which they start to fire action potentials spontaneously. They also start to receive synaptic inputs as cortical network matures, which drive, boost and modulate firing activity of the developing cortical neurons. This neuronal activity, “spontaneously” generated in the developing neurons and cortical network, has been thought to contribute to the formation of connections in the cortex during the developmental period before sensory inputs come in [28, 32, 33, 45, 86].

Garaschuk and colleagues used Ca^2+^ imaging technique to monitor neuronal activity in the developing cortical circuits in a slice preparation [42], and later in the intact brain [43, 49]. They found that spontaneous oscillatory Ca^2+^ waves traveled across cortical slices taken from P1–P4 rats and named them cortical early network oscillations (cENOs). cENOs were typically observed once per 1–12 minutes, and many neurons (typically over 80% of the neurons in the recorded area) participated in the wave. cENOs were completely blocked by AMPA-and NMDA-type glutamate receptor antagonists but not by GABA receptor antagonists, suggesting that cENOs are driven by glutamatergic transmission. Later, by using a similar approach, Allène and colleagues reported another synapse-driven network pattern in neonatal cortical slices, named giant depolarizing potentials (GDPs) [46]. GDPs are different from cENOs, in that they are driven by GABAergic transmission, occur at a higher frequency, recruit smaller number and more localized population of neurons. In addition, GDPs emerge at later stages in cortical development than cENOs (P5–9 for GDPs versus P1–4 for cENOs). These differences may suggest that cENOs and GDPs are involved in different aspects/phases of cortical circuit formation. Ca^2+^ waves have been observed in vivo rat/mouse cerebral cortex [43, 48–50], but these studies did not determine whether they corresponded to cENOs or GDPs.

Correlated Ca^2+^ activity mentioned above mostly reflects neuronal firings [46]. Extracellular recordings have detected similar network activities in neonatal rat/mouse cerebral cortex [44, 47, 76, 87, 88]. Yang and colleagues reported three distinct patterns of synchronized oscillatory activity in neonatal rat cortex: spindle-bursts, gamma oscillations, and long oscillations [47]. Spindle-bursts are neuronal burst firings of 1-2 s in duration, ~10 Hz in frequency, and observed approximately every 10 s. Gamma oscillations are neuronal activities at a frequency of 30–40 Hz, duration of 150–300 ms, and occur every 10–30 s. Spindle-bursts and gamma oscillations do not propagate but synchronize a local cortical network. In contrast, long oscillations propagate over large cortical regions. They occur every 20 m, last >40 s, and synchronize in the 10–20 Hz frequency range over 600–800 *μ*m. The precise relationship between the two types of Ca^2+^ waves (cENOs and GDPs) and the three types of electrical activities (spindle-bursts, gamma oscillations, and long oscillations) is to be clarified.

All three types of electrical activity can be elicited by activation of the periphery. For example, in the somatosensory cortex, tactile, or electrical stimulation of whiskers can induce these network activities [47]. It has also been shown that spontaneously generated correlated activity in the retina (retinal waves) is transmitted to induce spindle-bursts in the visual cortex [76]. However, blocking the peripheral inputs can only reduce, but not eliminate, these network activities, suggesting that the peripheral inputs are not the only mechanism to trigger these activities [50, 76]. Spindle-bursts are modulated by cholinergic inputs [89], and microcircuits between cortical neurons and subplate neurons (a transient population of neurons that resides in the neonatal cortical white matter) play a critical role in the generation of spindle-bursts [88, 90–92].

Interestingly, spindle-bursts and gamma oscillations are sometimes synchronized between hemispheres [47]. In this experiment, multielectrodes were inserted into both hemispheres simultaneously, and network activity recorded in each hemisphere was compared. The amount of interhemispheric synchronization increases progressively from P0 to P7, parallel to the development of callosal connections. It is not clear whether this synchronization occurs via callosal connections nor whether these activities can travel between hemispheres though callosal axons; however, surgical transection of the corpus callosum in neonatal rats modulates the expression of spindle-bursts [87], suggesting an existence of interhemispheric communication at this early stage. The synchronized neuronal activities between hemispheres may play a role in the formation and maturation of callosal connections.

The activity of individual cortical neurons and cortical network can be modulated by environmental factors. For example, the emergence of cENOs and GDPs is influenced by experimental conditions such as anoxia and aglycemia [46]. Some cortical network activity during neonatal periods is shown to be influenced by the hormone oxytocin, which is released by the mother during delivery [46, 93]. Alterations in the activity of individual neurons and network caused by these environmental factors may impede activity-dependent circuit formation in the cortex, including callosal axon projections.

The activity of cortical neurons and network can also be modulated by genetic factors. For example, genetic mutations in ion channels may affect excitability of neurons, causing some diseases such as epilepsy. KCNQ2 is a type of K^+^ channels crucial for the regulation of excitability in cortical neurons, and its genetic mutations are responsible for neonatal epilepsy (benign familial neonatal convulsions: BFNC) [95–99]. All disease-causing mutations in KCNQ2 identified so far result in loss-of-function of channel activity [100]. Transgenic expression of a dominant-negative KCNQ2 mutant channel in developing mouse cerebral cortex is shown to induce spontaneous seizures [94]. Another study shows that a mouse model of human KCNQ2 mutation for BFNC exhibits early onset spontaneous seizures, reminiscent of the phenotype in human patients [101]. In both studies, reduced KCNQ2 channel activity resulted in abnormal cortical activity as recorded by electroencephalogram (EEG) or electrocorticogram (ECoG). Dysfunction of KCNQ2 during the first postnatal week induces morphological changes in the hippocampus [94], implying that repeated seizures during the neonatal period have adverse effects on cortical circuit formation [102, 103].

## 4. Do Mutations in Ion Channels Affect Activity-Dependent Callosal Connection Formation?

**Figure 3:**
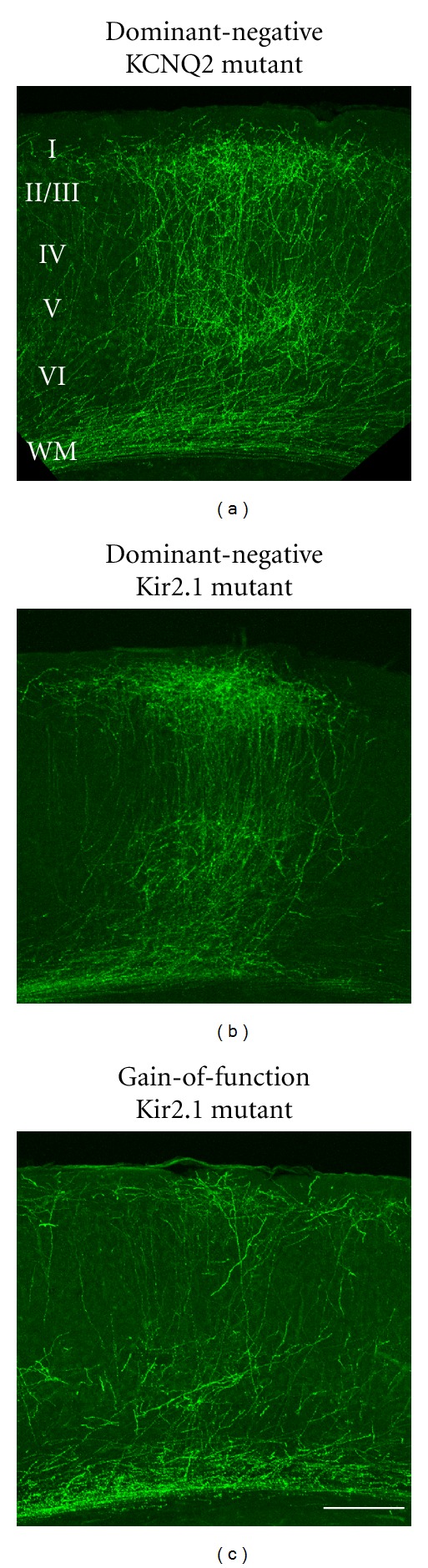
Effects of expression of several ion channels on callosal axon projections. (a) Expression of a dominant-negative KCNQ2 K^+^ channel [94] does not influence callosal axon development. (b) A disease-causing, dominant-negative Kir2.1 mutant does not impair callosal axon projections. (c) Expression of a gain-of-function Kir2.1 mutant in callosal projection neurons impedes their axonal projections. Scale bar, 200 *μ*m. Adapted from [38].

To test the idea that activity-dependent callosal axon projections may be affected by mutations in ion channels, we examined the effect of expression of a dominant-negative KCNQ2 mutant channel on callosal axon projections. As shown in Figure 3(a), we observed no apparent defects in overall projection pattern of callosal axons. We also examined the effect of expression of several disease-causing mutant Kir2.1 channels on callosal axon projections [38]. Kir2.1 is expressed in cardiac myocytes as well as cortical neurons, and its genetic mutations are responsible for Andersen syndrome, a disease associated with periodic skeletal muscle paralysis and cardiac arrhythmia [104, 105]. Most of these mutations result in loss-of-function with dominant-negative suppression of channel activity [105, 106]. Because reduced Kir2.1 channel activity causes severe manifestations in skeletal and cardiac muscle, we wondered if it might also affect circuit formation in the cerebral cortex. No apparent defects were observed in the development and axonal projection pattern of callosal neurons expressing disease-causing, dominant-negative Kir2.1 mutants [38] (Figure 3(b)). However, we found that a gain-of-function mutation in Kir2.1 (V93I), associated with familial atrial fibrillation (a cardiac disease characterized by rapid and irregular activation of the atrium) [107] caused severe defects in callosal axon projections [38] (Figure 3(c)). It has not been reported that patients with this Kir2.1 mutation have brain phenotypes [107]. However, there are some cases where a single mutation in an ion channel expressed in both heart and brain (e.g., KCNH2 and KCNQ1) can cause abnormalities in both tissues (cardiac and neural channelopathy) [108–110]. Kir2.1 is expressed in both cardiac myocytes and cortical neurons, and enhanced Kir2.1 activity can have deleterious effects on callosal connection formation. It is therefore possible that anatomical and functional assessment may reveal some abnormality in the structure and/or function of the corpus callosum in patients with the Kir2.1 gain-of-function mutation.

## 5. Concluding Remarks

Callosal connections mediate interhemispheric communication. They serve to integrate and coordinate information between hemispheres, thus involved in higher cognitive functions. Malformations such as partial or complete agenesis of the corpus callosum are associated with many human congenital disorders [18, 111], and significant reductions in its size are frequently reported in patients with certain psychiatric and developmental disorders [112–117]. It is important to identify factors affecting function, structure, and development of the corpus callosum. 

During development of callosal connections, both activity independent and dependent mechanisms are involved. Many genetic factors responsible for the activity independent processes have been reported [18], but “activity-dependent factors” have not been identified.

Recent advancements in electrophysiological and Ca^2+^ imaging techniques have enabled us to monitor neuronal activity in neonatal cerebral cortical circuits. These new techniques will be useful to examine how cortical activities are modulated by genetic and environmental factors. Hypo-or hyperactivity in neonatal cortical circuits caused by these factors may induce abnormality in the cortical architecture including the corpus callosum. In addition, genetic techniques such as in utero electroporation will allow us to identify molecular signals critical for activity-dependent callosal axon projections. Future work would link the factors that disturb activity-dependent callosal connection formation, with those that influence the patterns of neuronal activity in the developing cortex.
